# Predictive pollen-based biome modeling using machine learning

**DOI:** 10.1371/journal.pone.0202214

**Published:** 2018-08-23

**Authors:** Magdalena K. Sobol, Sarah A. Finkelstein

**Affiliations:** Department of Earth Sciences, University of Toronto, Toronto, Canada; The University of Sydney, AUSTRALIA

## Abstract

This paper investigates suitability of supervised machine learning classification methods for classification of biomes using pollen datasets. We assign modern pollen samples from Africa and Arabia to five biome classes using a previously published African pollen dataset and a global ecosystem classification scheme. To test the applicability of traditional and machine-learning based classification models for the task of biome prediction from high dimensional modern pollen data, we train a total of eight classification models, including Linear Discriminant Analysis, Logistic Regression, Naïve Bayes, K-Nearest Neighbors, Classification Decision Tree, Random Forest, Neural Network, and Support Vector Machine. The ability of each model to predict biomes from pollen data is statistically tested on an independent test set. The Random Forest classifier outperforms other models in its ability correctly classify biomes given pollen data. Out of the eight models, the Random Forest classifier scores highest on all of the metrics used for model evaluations and is able to predict four out of five biome classes to high degree of accuracy, including arid, montane, tropical and subtropical closed and open systems, e.g. forests and savanna/grassland. The model has the potential for accurate reconstructions of past biomes and awaits application to fossil pollen sequences. The Random Forest model may be used to investigate vegetation changes on both long and short time scales, e.g. during glacial and interglacial cycles, or more recent and abrupt climatic anomalies like the African Humid Period. Such applications may contribute to a better understanding of past shifts in vegetation cover and ultimately provide valuable information on drivers of climate change.

## Introduction

Past environmental conditions can be inferred from proxy data such as pollen. Studies of fossil pollen have been instrumental in our understanding of past shifts in vegetation [1,2] and variations in climate [3–5]. The accuracy of pollen-based paleoenvironmental reconstructions is dependent on numerically quantified relationships between modern pollen assemblages and variables of interest, be they quantitative or qualitative. These calibration sets allow for robust numerical modeling of pollen-vegetation-climate relationships. Thus, meaningful estimates of past environments rely on large and accurate modern calibration sets [6].

Over the last decades, pollen data have been most frequently used in quantitative reconstructions of climate variables [7–12]. However, there are few models utilizing complete pollen datasets for prediction of discrete variables such as large scale vegetation assemblages i.e. biomes. For reconstructions of past biomes from fossil pollen data, there has been one particularly prominent approach. The biomization method decomposes a biome's floral complexity to a few representative taxa using a plant functional types (PFTs) approach which assumes that a plant's form and function are related [13,14]. This functional relationship may be used as a reliable substitute for biomes. In two separate steps, the biomization method assigns pollen taxa to one or more PFTs, and PFTs to one or more biomes resulting in two matrices. To arrive at the final biome-taxon matrix, binary matrix multiplication is performed on the two matrices resulting in the assignment of pollen taxa to biomes. Pollen samples are assigned to biomes using fuzzy logic. The biomization technique is the primary method for predicting biomes from pollen data and for reconstructing past biomes. Since its development, this method has been embraced by paleoecologists and applied to fossil pollen sequences across the globe to model shifts in the distribution of past biomes [15–18].

Nonetheless, the biomization method uses only a subset of pollen taxa to make predictions. By selecting only a few taxa to characterize biomes, complex associations between and interactions among contributing factors may be neglected. Such data exclusion may potentially lead to information loss that propagates into overly simplistic interpretations, particularly when applied to fossil proxy assemblage for past reconstructions. To improve results and interpretations of pollen-based paleoenvironmantal reconstructions we identify and stress the need for utilizing more complete pollen datasets, i.e. datasets that are not excessively manipulated by excluding certain pollen taxa such as aquatics, local or regional taxa.

This paper examines machine learning methods for the task of biome prediction using complete sets of pollen taxonomic data. Pollen-based biome modeling assumes that a given biome will impart its characteristic patterns in pollen data. As these patterns may be exceedingly complex, biome modeling using more complete pollen datasets is a complicated task; not only are pollen datasets challenging to analyze due to their high dimensional nature, the potential correlations between and complex interactions among pollen taxa are difficult to unravel. Furthermore, the possible number of combinations increases exponentially in high dimensional data [19] such as pollen datasets. As such, analyses of high dimensional data require large number of data points to extract a meaningful signal. In addition, adequate analytical tools must be available to identify patterns in pollen datasets. Detecting and recognizing patterns in complex datasets has been made possible due to increases in computational power and developments in machine learning fields focused on classification and prediction approaches.

### Objectives

In this paper we explore the ability of different statistical tool to predict biomes from African and Arabian surface pollen data.

The objectives of this paper are to: 1) review machine learning classification methods suitable for prediction of biomes using pollen datasets; 2) test the applicability of supervised machine learning classification models for the task of biome prediction from more complete modern pollen data given a set of training examples of *a priori* labeled observation set; 3) analyze and statistically compare chosen classification methods; 4) identify, using statistical measures, the highest performing classification model able to accurately predict biomes from modern pollen data; 5) qualitatively compare our best ML-based model against the classical biomization method previously developed for the region.

## Materials and methods

Supervised classification is an inductive learning process wherein knowledge gained from examples can be used to generalize a discrete-valued mapping function separating data into different categories [20]. The two most common types of classification are binary and multi-class classification. In binary classification, the value for the prediction task is one of two discrete values. In the context of pollen-based predictions, an example of a binary classification task would be prediction of terrestrial vs marine context for a pollen assemblage. In multi-class classification, the value of interest is one of a set of discrete values. Prediction of multiple biomes from pollen data is an example of multi-class classification. Multi-class classification may be re-framed into a simpler binary classification via an approach known as *one-versus-rest* [21] wherein separate classification models are fitted for individual biome class against the rest of the biome classes combined.

Datasets used for supervised classification typically consists of a list of *examples*, each of which consists of a set of *features* and a target *label*. In the case of pollen datasets, the samples comprise the examples while individual pollen taxon abundances represent the features. In supervised classification each instance in the dataset has a biome assigned to it by a human; these assignments are called *labels* and represent example target values being predicted. For training and evaluation, the original dataset is divided into two sets. A *training set* is a larger complement of the original dataset used to estimate parameters for a model. A *test set* is a smaller portion of the original dataset reserved for evaluation of the model on previously unseen and unlabeled data. The procedure during which an algorithm learns parameters specific to a particular model using the training set is called *model fitting*.

How accurately a model predicts biome labels from the training data depends in large part on the configuration variables of the training process. These *hyper-parameters* are unique to each model. The optimal set of hyper-parameters may be identified through *cross validation* by further splitting the training set into training and validation sets where hyper-parameters are chosen based on the classification performance on the validation set. The outcome of the model fitting is a trained model.

The predictive performance of a trained model is tested on the reserved test set. Results can be reported in a *confusion matrix* where the model predictions of biomes for the test examples are displayed against their true and known biome labels. The confusion matrix provides numerical summaries on correct and erroneous classifications made by a model. In the case of binary classification, the classes are typically defined as true or false. *True positives* (TP) and *true negatives* (TN) both represent correct classifications by a model. While TP indicates an example correctly assigned to its true label, the TN indicated a correct classification of false example. Errors fall into one of two categories: 1) when a model assigns an example to the true class where the known label is false; this type of error is known as the *false positive* (FP), commonly referred to as type I error; and 2) when an example is known to be true yet the model does not predicts a true label; this is known as a *false negative* (FN) or type II error [21]. These terms can be generalized to multi-class classification using the one-vs-rest approach.

From the confusion matrix, a number of *evaluation methods* may be calculated to measure how well a model performs on previously unseen data. Model *accuracy*, or proportion correctly classified, is a measure of a how often the model's prediction is correct. Accuracy on a test set is calculated as (TP + TN)/(TP + TN + FP + FN). *Recall*, or the true positive rate, measures the model's ability to detect the positives. Recall is calculated as TP/(TP + FN). To assess how many of the positively classified examples were relevant, positive predictive rate, or *precision*, is calculated as TP/(TP + FP). These two metrics can be used to calculate the *F1* statistic, the harmonic mean of recall and precision. *Cohen's kappa* statistic is a measure of unbiased models' performances taking into account imbalances in class distributions. Kappa provides a measure of a model's predictive performance as compared to the performance of the model achieved by random chance [22].

In addition, summary analyses for classification models are available. One of the most informative and widely used types of summary analyses are *feature importances* [23]. The contribution of individual pollen taxa to the overall prediction accuracy of a model is measured via the *Mean Decrease in Accuracy (MDA)*. The MDA measures how much of a model's test accuracy is degraded by randomly permuting the values of a given feature. An MDA value of zero represents a feature not used in the prediction while a feature with higher values indicate that the model was relying heavily on that feature for prediction [23].

Lastly, in prediction modeling two important sources of model error are *bias* and *variance* [23]. Bias is a product of a model's assumptions about the distribution of data. Bias increases for models with strong assumptions. As a result, such models will fit data into assumptions whether or not the data actually conforms to those assumptions. On the other hand, models which hold few assumptions about the data distribution, and can learn more complicated relationships between the features and labels, often have high variance. Model variance stems from the combination of the model's predictive power and sampling error. Thus, characteristics of a training set affect the parameters of the learned function as the model is able to *overfit* to the sampling noise in the training data. Models with high variance are referred to as *unstable* [21]. Supervised machine learning algorithms aim to decrease both bias and variance to achieve higher predictive power. One way to reduce a model’s bias and variance may be achieved via hyper-parameter optimization [23]. In summary, a robust model optimized by hyper-parameter tuning is characterized by high scores on the evaluation metrics on the test set implying low model bias and variance.

### Materials

For model training, we use a collection of published modern pollen data from Africa (previously stored at http://medias.meteo.fr/) [24] which we assign to biome types using the world terrestrial ecosystem classification (Table 1) [25,26]. From the original 1198 modern pollen samples, 73 were excluded due to a lack of coordinates or inappropriate context (marine).The samples were collected from a range of contexts including surface (733), lakes (243), rivers (75), traps (48), middens (25), and ice (1) that represent nine biomes. The resulting dataset has 1125 biome examples described in terms of 119 pollen predictors.

**Table 1:** African biomes represented in the modern pollen data organized by biome, number of representative modern pollen samples, biogeographic region, and country.

Biome	Pollen	Biogeographic region	Country
Deserts and Xeric Shrublands	239	Namib and Karoo deserts and shrublands	South Africa, Namibia
		Kaokoveld Desert	Namibia, Angola
		Madagascar Spiny Desert	Madagascar
		Horn of Africa deserts	Somalia
		Socotra Island Desert	Yemen
Flooded Grasslands and Savannas	21	Sahelian flooded savannas	Mali, Chad, Niger, Nigeria, Cameroon, Senegal, Mauritania
		Zambezian flooded savannas	Botswana, Namibia, Angola, Zambia, Malawi, Mozambique
		Sudd flooded grasslands	Sudan, Ethiopia
Montane Grasslands and Shrublands	120	East African moorlands	Kenya, Tanzania, Uganda, D.R. Congo, Rwanda
		Ethiopian Highlands	Somalia, Eritrea, Sudan
		Zambezian montane savannas and woodlands	South Africa, Lesotho, Swaziland
Tropical and Subtropical Grasslands, Savannas, and Shrublands	415	Angolan Escarpment woodlands	Angola
		Zambezian woodlands and savannas	Zambia, Tanzania, Malawi, Zimbabwe, Mozambique, Angola, Namibia, Botswana, D.R. Congo, Burundi
		Sudanian savannas	Central African Republic, Chad, Uganda, Ethiopia, D.R. Congo, Cameroon, Sudan, Nigeria, Eritrea
		East African acacia savannas	Kenya, Tanzania, Sudan, Ethiopia, Uganda
Tropical and Subtropical Moist Broadleaf Forests	314	Madagascar moist forests	Madagascar
		Guinean moist forests	Ghana, Guinea, Côte d’Ivoire, Liberia, Sierra Leone, Togo
		Eastern Arc montane forests	Tanzania, Kenya
		East African coastal forests	Tanzania, Kenya, Mozambique, Somalia
		Albertine Rift highland forests	D.R. Congo, Rwanda, Uganda, Burundi, Tanzania
		East African highland forests	Kenya, Tanzania, Uganda
		Seychelles and Mascarene Islands forests	Mauritius, Seychelles, Comoros, Reunion, Rodrigues
		Gulf of Guinea Islands forests	São Tomé and Príncipe, Equatorial Guinea,
		Macaronesian forests	Azores, Madeira, Canary, Cape Verde Islands
		Congolian coastal forests	Cameroon, Gabon, R. Congo, Nigeria, Equatorial Guinea, Benin
		Western Congo Basin forests	Central African Republic, Cameroon, R. Congo, Gabon, D.R. Congo, Equatorial Guinea
		Northeastern Congo Basin forests	D.R. Congo, Central African Republic, Sudan, Uganda
		Southern Congo Basin forests	D.R. Congo, Congo, Angola

### Methods

**Fig 1:**
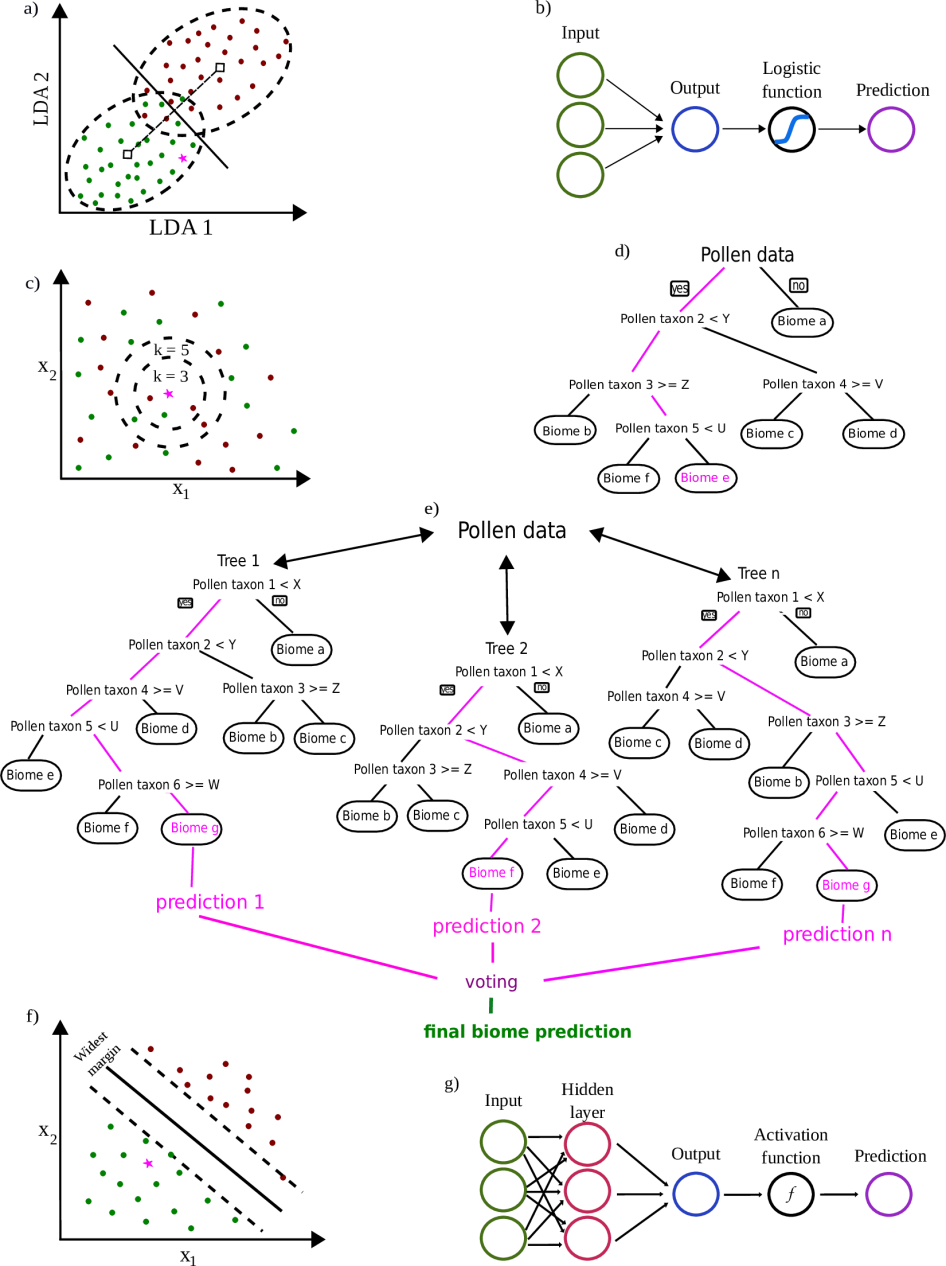
Simplified representation of the classification process for the statistical and machine learning algorithms used for predicting biome. a) Linear Discriminant Analysis, b) Logistic Regression, c) K-Nearest Neighbors, d) Classification Decision Tree, e) Random Forest, f) Support Vector Machines, and g) Neural Networks. Naïve Bayes classifier not depicted. Red and green dots in panels a), c), and f) represents two classes of data while pink stars represent a new pollen assemblage without biome label. Pink lines in d), and e) represent decision paths.

We examine a number of different supervised classification models for the task of predictive biome modeling using modern pollen data. The models were chosen on the basis of their suitability for ecological and paleoecological application [27,28], and specifically for: 1) multivariate and high dimensional pollen data, and 2) classification of more than two biomes classes, i.e. multi-class classification. Models considered here represent parametric, semi-parametric and non-parametric supervised machine learning classification methods (Fig 1).

In addition to the assumptions underlying the learning process, models may be further distinguished by the rules used for separating classes of biomes and data transformations required. To separate different classes of data into discrete categories, models use rules that specify how to assign a given modern pollen assemblage to a biome type; depending on whether a given example fits the conditions it may be included in or excluded from a particular class. These decision rules may be divided into linear and non-linear rules [23]. A linear classifier is defined by linear decision boundaries, such as straight lines or planes, used to separate different groups of data. On the other hand, decision rules employed by non-linear classification models may take any form, for example yes/no questions or non-linear shapes represented by a sigmoid function or radius of a circle. Frequently, the original data may be challenging for any given model to correctly separate into groups. Therefore, transformations of the original data may be necessary to facilitate clear distinctions between classes of data.

The classification process of each model considered here is reviewed below.

### Parametric classification models

In parametric classification, the learned mapping function has a known form with a fixed number of parameters [21]. This type of learning process is computationally fast and conceptually easy to understand. Furthermore, parametric learning methods do not require great amount of data to learn the mapping function. However, if the model assumptions do not fit the actual data distribution, parametric models may be characterized by high bias [23]. As a result, the predictive capacity of these methods is fixed, constraining their ability to detect complex patterns in the data. This is particularly the case with the linear types of classification methods. Thus, linear parametric models are generally better suited to simpler problems. Examples of early linear parametric classification methods are linear discriminant analysis and logistic regression.

#### Linear discriminant analysis

Linear discriminant analysis (LDA henceforth) is a standard classification tool [29] frequently used in paleoecology for data visualization and dimensionality reduction [30]. LDA as a classification method finds linear combinations of pollen features that best separate classes of data into groups.

In a multi-class classification problem, LDA locates a central point to all pollen data. The distance between the central point and points central to each biome category is measured using the Mahalanobis metric [31]. Groups of biomes are separated by straight lines on the basis of the maximized distance between each biome category and center point, and minimized scatter for each biome category [21]. A new unlabeled example is classified by LDA to a particular biome type by calculating its distance to the biome categories (Fig 1A).

LDA has found uses in a variety of climate related research including application to geochemical data for classification of paleo-sediments [32], modeling future precipitation and storm days [33], and predicting occurrence of various landslide types [34].

#### Logistic regression

Logistic regression (LR henceforth) is a statistical tool for estimating the probability of categorical dependent variables [35,36]. LR predicts biome class by calculating a weighted sum of the input data, in our case pollen abundances, and a constant bias term. The logistic function transforms this weighted sum into a probability by compressing it into the range between zero and one [20]. The output of the logistic defines a linear decision boundary used to separate biome classes and assigns observations to biome classes depending on which side of the line they fall (Fig 1B). In addition to one-vs-rest, LR may be generalized to multi-class classification using multinomial LR (mLR). In the multi-class problem, a single mLR model is trained for all biome classes to estimate the probability of a given sample belonging to each biome class.

Examples of the LR classifier uses in ecology and paleoecology include prediction of presence of different tundra vegetation types [37], prediction of probability of fire occurrences [38], and identification of groups of Early Jurassic plants from fossil data [39].

#### Naïve bayes

Another linear classification method suitable for data with high number of features is the Naïve Bayes (NB henceforth). Based on the Bayes' theorem [40,41], the model assumes conditional independence between predictor variables given the label. This assumption greatly reduces the complexity of highly dimensional datasets.

During training, the proportion of biome classes (*P(biome)*) in the training set is calculated along with the probability of a each pollen taxon conditional on the biome class (*P(taxon|biome)*). For example, given a dataset of 100 sites where the number of grassland sites is 20, the proportion of grassland would be 0.2. If the Euphorbiaceae family appears in 15 of those 20 grassland sites, the conditional probability of Euphorbiaceae given grassland equals 0.75.

During test time, the continuous values for pollen features of the unlabeled example are transformed into likelihood tables. The Bayes equation is used to calculate probability for each biome class [23]. A biome class with the highest probability is considered the most likely and becomes the final prediction for the unlabeled instance.

The NB model has been applied to problems in ecology, environmental research and geosciences such as modeling species distributions [42], assessing flood risks [43] and water quality [44], and mineral mapping [45]. In palynology, the NB algorithm has been applied to automated pollen recognition [46].

### Non-parametric classification models

Non-parametric learning methods make fewer assumptions about the underlying function than their parametric counterparts. Furthermore, the number of model parameters is not finite or bounded as they are in parametric methods. As a result, the capacity of non-parametric models for accurate predictions increases with increasing data. The superior classification performance of the non-parametric models comes at a cost as their performance is dependent on the amount of data available, i.e. more data ensures a better performance. Furthermore, training of some non-parametric models may require more computational power and time than training parametric models. Lastly, interpretations of results may be more difficult due to a high degree of stochasticity inherent in some of the more complex non-parametric models [21].

#### K-Nearest neighbors

One of the earliest non-parametric methods is the K-Nearest Neighbors (KNN henceforth). This model is well known to paleoecologists in its quantitative incarnation as the Modern Analogue Technique (MAT) [47] for prediction of continuous climate variables from fossil pollen [48,12] and other proxy data such as diatoms [49] and dinoflagellate cysts [50].

The KNN model stores all the examples from the training set shown during training time. When an unlabeled example is presented at prediction time, the KNN classifier searches for a defined number (K) of nearest cases most similar to the new example using a similarity function. The labels for the nearest neighbors are retrieved and the biome label for the new example is assigned to the class most common among the nearest neighbors (Fig 1C) using a majority vote rule [30]. The KNN classifier is relatively simple and easy to understand lending itself well as a benchmark for comparison to other methods.

#### Classification decision trees

Classification trees [51] are popular non-parametric machine learning algorithms for classification and regression predictive modeling. The goal of a decision tree is to accurately split a dataset into groups in the fewest steps possible. The classification decision tree (CDT henceforth) achieves this by learning a series of explicit if-then rules on features resulting in a decision process that predicts an outcome. Pollen proportions are used to answer a series of increasingly precise yes/no questions to categorize a biome type. For example, when dealing with continuous values such as pollen percentages, questions asked at nodes involve threshold percentages of a pollen taxon chosen as the best variable to perform a split at that node.

During training time, pollen data are split using the best pollen taxon such that the data assigned to the resulting two daughter nodes retain maximum heterogeneity between themselves and maximum homogeneity within themselves [23]. The splitting process continues iteratively until the remaining subsets of pollen data are classified and the leaves contain the same or dominant majority of a biome type. This process frequently produces a function that is closely fit to the training data often resulting in an overly complex model that is unable to generalize well during test time. To improve its prediction, the unnecessary complexity of the tree may be reduced by cutting back a tree to the point of minimal cross-validation error [23].

During prediction time, a new unlabeled example is run through the established sequence of rules. Starting at the top of the tree, a decision is taken at each level based on the appropriate pollen proportion until it reaches a leaf, or terminal, node. The prediction for the new unlabeled example is the biome label associated the leaf node (Fig 1D).

The CDT model is relatively easy to interpret. The visualization of a decision tree shows the exact decision process behind every prediction. However, CDTs are prone to over-fitting to the training data by adding more rules to arrive at precise classification of data. In effect, the algorithm memorizes the training data leading to poor prediction for a new and previously unseen example. In other words, CDTs have low bias and high variance. This high variance of CDTs is a product of the hierarchical nature of the algorithm as the top-down learning process in a decision tree propagates potential errors down the tree [21].

Decision trees have been applied to ecological problems [52] such as prediction of habitat [53]. In paleoecology, CDTs often serve as tools for identification of diagnostic morphological features, for example in leaf stomata [54] or diatoms [55].

#### Random forest

Next, we consider an algorithm that addresses the problem of the variance-bias trade-off in decision trees. Random Forest (RF henceforth) is an ensemble of individual decision trees fully grown in a similar manner as trees in CDT [56]. However, two randomization steps in the learning process make the RF more robust than an individual decision tree. Unlike the CDT model, pollen data used for building each tree in RF is randomly subsampled from the original dataset. Furthermore, nodes of individual trees in the RF are also split using a random subsample of pollen features from the original dataset [57]. When a new unlabeled example is presented to RF, it is run through all trees in the forest (Fig 1E). Each tree provides a prediction of biome types for the new example. The predictions are then averaged across all trees and the biome type with the highest probability as identified by the majority vote rule becomes the final prediction for the given example [23].

The RFs algorithm is one of the highest performing non-parametric classifiers and has found successful application in ecology for modeling future species distribution under various climate scenarios [58], prediction of rare and invasive species [24], as well as classification of land cover [59,60], and savanna trees from hyperspectral and LiDAR data [61]. In paleoecology, RFs have not been as widely utilized with only some application to regression problems [62] and modeling of past vegetation [28].

#### Support vector machines

The final supervised non-parametric machine learning classification model considered for biome prediction using pollen data is Support Vector Machines (SVMs henceforth). SVMs transform original data into a new higher dimension feature space such that the transformed features are easier to separate using a linear classifier [63,64]. SVMs calculate the similarity between two points in the original feature space for the corresponding points in the transformed feature spaces. The similarity measure between data points in the transformed feature space is referred to as a *kernel function* or simply a *kernel* [20]. The resulting groups are separated with planes using a method characteristics to SVMs called the *maximum margin separator* or the *widest street approach* [20]. A hyper-plane is drawn in kernel space between biome classes such that the margin of the decision boundary between the two closest data points of each biome class is maximized (Fig 1F). As in LR, an unlabeled pollen assemblage is classified based on which side of the decision boundary they fall. However, for SVMs this determination is done in kernel space.

The SVMs have been widely applied to ecological problems such as modeling of species' niche [65], prediction of plant pathogens [66] and ground water [67], mapping vegetation [68,69], and classification of aquatic species [70]. The application of SVMs to paleoecological problems have been more slow coming and the algorithm does not appears to be particularly suitable for climate reconstruction [71]. However, SVMs show potential for recognition and classification of pollen grains [72].

### Semi-parametric classification model

Lastly, we consider a model from the semi-parametric domain of machine learning classifiers. Semi-parametric methods combine features of parameteric and non-parametric approaches. For example, some semi-parametric models have parameters that are learned during training but do not make assumptions about the form of the function. As a result, semi-parametric methods are often able to model more complicated relationships between predictor features and class labels.

#### Neural networks

In the semi-parametric machine learning domain, we consider Neural Networks (NN henceforth), one of the most powerful machine learning algorithms currently available [73]. The NN model is a generalization of the previously discussed LR with the addition of extra computational steps between the input feature and the output class labels. These steps, or *hidden layers*, result in a learned non-linear transformation of the data. This new representation is then passed through the logistic function to obtain final classification; thus, LR may be interpreted as a special case of NN (Fig 1G).

The simplest NN model has one hidden layer which is a collection of hidden units that compute new representation of the original data. The value, or *activation*, of each hidden unit is calculated by the weighted sum of the input features passed through a non-linear *activation function* (e.g. rectified linear, sigmoid, tanh). The output values of the hidden units are combined to calculate another weighted sum which is then transformed by a logistic function for the final prediction. Training a NN model involves finding an optimal set of weights; this is done by minimizing the error, or *loss*, over the training data via gradient descent using the back-propagation algorithm [74]. Here, the loss function is a smooth function which measures how different the model's prediction is from the ground truth of the training data.

Neural Networks have been widely applied to ecological problems such as pollen classification in honey products [75], time-series analysis to investigate climate drivers in subalpine forests [76], weather forecasting [77], modeling non-linear relationships in aquatic ecology [78] and future warming [79], predicting species distribution [80] and water resources [81]. For paleoecology, the NNs are a promising approach for automated pollen grain recognition that would aid in the pollen identification process [82]. In addition, NNs have been used in paleoecological research for classification of indicator species [83], estimating paleo-salinity changes in sea surface water [84], and pollen-based quantitative climate reconstructions [85,86].

### Model training

**Fig 2:**
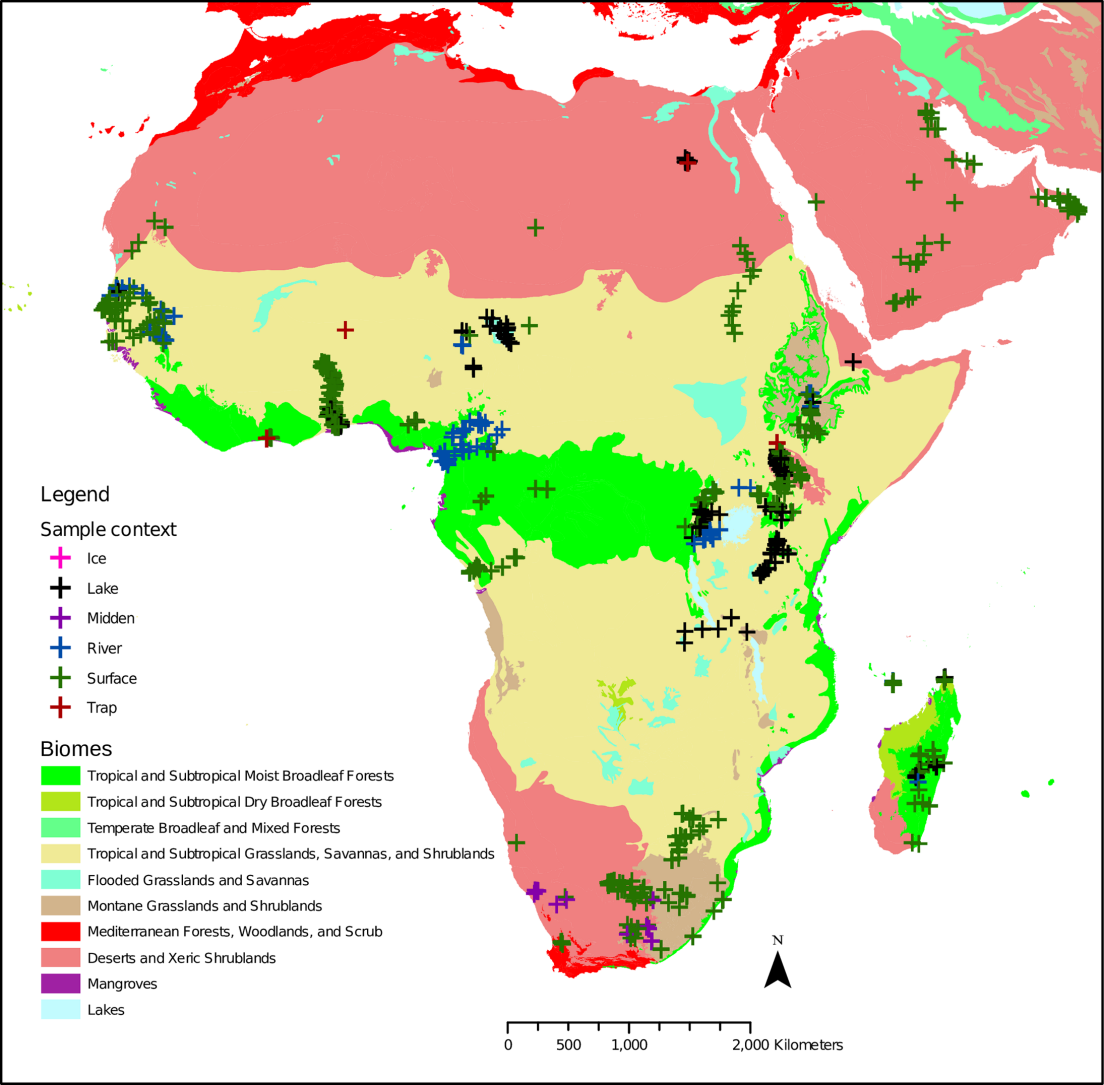
Distribution of modern pollen samples (Gajewski et al., 2002) across African biomes (Olson et al., 2001).

The assignment of modern pollen samples to biome classes based on the world terrestrial ecosystem classification of Olson et al. (2001) was carried out in ArcGIS 10.4 where the cell values from the imported vegetation map were extracted for each pollen data point. Pollen points that did not fall within a biome (i.e. lake) were manually labeled to the nearest biome by the author. The 1125 modern pollen samples represent the following nine biomes (Fig 2): Deserts and Xeric Shrublands (239), Flooded Grasslands and Savannas (21), Mangroves (3), Mediterranean Forests, Woodlands, and Scrub (4) Montane Grasslands and Shrublands (120), Temperate Grasslands, Savannas, and Shrublands (8), Tropical and Subtropical Dry Broadleaf Forests (1), Tropical and Subtropical Grasslands, Savannas, and Shrublands (415), Tropical and Subtropical Moist Broadleaf Forests (314).

We trained eight machine learning classification models: Linear Discriminant Analysis, Logistic Regression, Naïve Bayes, K-Nearest Neighbors, Classification Decision Trees, Random Forests, Neural Networks, and Support Vector Machines. The analyses of the models were carried out in Python version 2.7.12 using the *scikit-learn* [87], *numpyc* [88], and *pandas* [89] packages. Data were preprocessed as follows; firstly, pollen abundance data were scaled to the range 0–1. Secondly, biomes represented by less than 10 sites were removed to improve statistical requirements for sample representativeness. Lastly, to increase the signal-to-noise ratio, we removed rare pollen taxa such that only taxa present above 3% in at least 1 site were kept [6].

The biome-labelled modern pollen dataset was divided into a training and test set in a 9:1 ratio and maintaining class distribution. The models were optimized using 50 iterations of random search for their respective hyper-parameters [90] and performing 10 fold cross-validation for each iteration of the search over training data only. Biome class imbalances were preserved during cross-validation to ensure that the training set of each fold retains the same class imbalances as the entire data set. For LDA we optimized over the solver (*svd*, *eigen*, and *lsqr*) and the number of n_components for dimensionality reduction (*1–5*). For the LR model we searched for: fit_intercept (*true* or *false*), class_weight (*none* or *balanced)*, regularization strength C (*0–1000*) and multi_class option (*ovr* or *multinomial)*. For the Naïve Bayes classifier we searched over additive smoothing parameter alpha (*0–1*), fit_prior (*true* or *false*) and the distributions form of the model (*multinomial*, *Gaussian*, and *Bernoulli*). For the KNN model we searched for the optimal number of neighbors n_neighbours (*1–10*), the weight function (*uniform* or *distance*), the algorithm (*ball_tree*, *kd_tree* or *brute*) for computing distances between neighbors, and power parameter for the Minkowski metric p (*1–4*). For both the CDT and RF models, we optimized over the following hyper-parameters: criterion (*gini* or *entropy*), max_features (*auto*, *sqrt*, *log2* or *none*), min sample split (*0–1*). Separately, for CDT we optimized over splitter (*best* or *random*), class weight (*balanced* or *none*), while for RF we search for n_estimators (*10–200*) and class_weight (*balanced* or *balanced_subsample)*. For the SVM algorithm we optimized over C (*0*.*001–100*), kernel *(rbf*, *poly*, *sigmoid)*, gamma (*0*.*001–1000*) degree *(1–3)*. For the NN classifier we optimized over the following hyper-parameters: hidden_layer_sizes (*50*, *100*, *200*), alpha (*0–0*.*1*), activation (*logitstic*, *tanh* or *relu*), batch_size (*32*, *64*, *128*), learning_rate (*constant* or *adaptive*), max_iter (*20–200*).

Models were then fitted to the entire training set using the best hyper-parameters as determined during random search. We examine the models' predictive performances on the test set using the following statistical measures: overall models' accuracy, kappa statistic, F1, and weighted precision and recall. The model scoring highest on these evaluation metrics, as determined by cross-validation, represents the best classifier. The highest performing model was evaluated separately by calculating the accuracy metrics on individual biome predictions for the test set.

Variable importances were calculated using the Mean Decrease in Accuracy (MDA) for each model to show the influence of individual pollen taxa to each model's predictions. Baseline accuracy was calculated for each trained model by testing performances on the reserved test set. Each pollen taxon in the test set was successively shuffled and then models were run again to calculate a change in accuracy. The shuffling procedure was repeated ten times and the mean was calculated to obtain the final MDA metrics.

For comparisons between results from our models and the biomization method, we provide new calculation of the corresponding evaluation metrics [17]. Precision, recall and F1 statistic are calculated from the original confusion matrix. Precision is calculated by dividing a given biome score by the sum of predicted biomes, or the column sum. Recall is calculated by dividing a given biome score by the sum of observed biomes, or the row sum. F1 statistic is a harmonic mean of the recall and precision. To calculate kappa statistic, the original confusion matrix [17], is converted into probabilities. Kappa is calculated from the probabilities by dividing the difference between the overall proportion of observed agreement (*P_o*) and the overall expected value of agreement due to chance (*P_e*) by *1 –P_e*. The *P_o* is calculated by adding the diagonal elements in the converted probability matrix. The *P_e* is calculated for each biome by taking the product of row and column sum for each biome and summing them.

## Results

After preprocessing the pollen and vegetation data, the total number of represented biomes was reduced from nine to five classes (Table 1): Deserts and Xeric Shrublands (239), Flooded Grasslands and Savannas (21), Montane Grasslands and Shrublands (120), Tropical and Subtropical Grasslands, Savannas, and Shrublands (415), Tropical and Subtropical Moist Broadleaf Forests (314).

Hyper-parameters found for each model and their respective values are listed in Table 2. Hyper-parameters for the majority of the models are optimized under three minutes on average (S1 Table). The most time demanding classifiers to optimize were the SVMs and NNs, while tuning of NB and LDA hyper-parameters was the fastest. Our pollen data labelled with biomes along with Python code are available as supplementary information, S2 Table, and S1 Python code respectively, as well as at GitHub.

**Table 2:** List of hyper-parameters identified for each model using random grid search (Bergstra & Bengio, 2012), their optimized values, and argument descriptions (Pedregosa et al., 2011).

Model	Parameter	Value	Argument description
LDA	n_components	3	Number of components for dimensionality reduction
	solver	svd	Solver to use
LR	multi_class	multinomial	Class type; either ‘one-versus-rest’ or ‘multinomial’
	C	973.755518841459	Inverse of regularization strength
	solver	lbfgs	Algorithm to use in the optimization problem
	fit_intercept	FALSE	Specifies if a constant should be added to the decision function
	class_weight	None	Weights associated with classes
NB	alpha	0.97375551884146	Smoothing parameter
	fit_prior	TRUE	Whether to learn class prior probabilities or not
	class_prior	None	Prior probabilities of the classes
KNN	n_neighbours	6	Number of neighbors to use
	weights	distance	Weight function used in prediction
	algorithm	brute	Algorithm used to compute the nearest neighbors
	p	1	Power parameter for the Minkowski metric
CDT	max_features	sqrt	Number of features to consider when looking for the best split
	min_samples_split	0.031313293	Minimum number of samples required to split internal node
	splitter	random	Strategy used to choose the split at each node
	criterion	entropy	Function measuring the quality of a split
	class_weight	None	Weights associated with classes
RF	max_features	sqrt	Number of features to consider when looking for the best split
	min_samples_split	0.007066305	Minimum number of samples required to split an internal node
	class_weight	balanced_subsample	Weights associated with classes
	criterion	entropy	Function measuring the quality of a split
	n_estimator	98	Number of trees in the forest
SVM	kernel	poly	Kernel type to be used in the algorithm
	C	21.234911067828	Penalty parameter C of the error term
	gamma	617.482509627716	Kernel coefficient
	degree	1	Degree of the polynomial kernel function
NN	hidden_layer_size	200	The n-th element representing the number of neurons in the n-th hidden layer
	alpha	0.017436642900	Regularization term
	activation	relu	Activation function for the hidden layer
	solver	adam	Solver for weight optimization
	batch_size	32	Size of minibatches for stochastic optimizers
	learning_rate	0.0001	Learning rate schedule for weight updates
	learning_rate_init	adaptive	The initial learning rate used
	max_iter	123	Maximum number of iterations

Models were fitted to 10 folds for each of 50 candidates, totaling 500 fits. Acronyms denote: LDA for Linear Discriminant Analysis, LR for Logistic Regression, NB for Naïve Bayes, SVM for Support Vector Machines, KNN for K-Nearest Neighbors, CDT for Classification Decision Tree, RF for Random Forest and NN for Neural Networks.

The highest performing model is RF, scoring highest on all evaluation metrics and achieving overall accuracy of 0.86 with a 0.85 precision and F1 scores on the test set (Table 3). The LR classifier is the second highest scoring model, closely followed by the NN model. The LDA, NB, SVM, KNN, and CDT models perform similarly to one another. Kappa measurement for RFs is highest (0.80) among the models considered, while the CDT and SVM classifiers have the lowest kappa values (0.66 and 0.67 respectively). The rage of kappa values (0.71–0.76) is similar among NB, KNN, and NN classifiers.

**Table 3:** Evaluation metrics calculated on the test set and reported in percent (%) for biome predictions for each classifier.

Evaluation Metric	Classification model							
	Logistic Regression	Linear Discriminant Analysis	Naive Bayes	Support Vector Machines	K-Nearest Neighbors	Decision Tree	Random Forests	Neural Networks
Accuracy	0.82	0.77	0.78	0.77	0.79	0.76	0.86	0.77
Precision	0.82	0.80	0.81	0.79	0.80	0.77	0.85	0.75
F1	0.81	0.79	0.78	0.75	0.79	0.75	0.85	0.76
Kappa	0.74	0.69	0.71	0.67	0.71	0.66	0.80	0.67

Recall foreach predicted vegetation type is calculated as the weighted number of correct predictions for a given known vegetation type. Precision for each predicted vegetation type is calculated as the weighted proportion of correctly classified vegetation unit to the sum of all predictions.

With the exception of one biome, the RF model scores high on evaluation metrics for predictions of individual biomes (Table 4). Scores for recall range between 0.73 and 0.93, for precision 0.83–0.92, F1 0.81–0.9, and kappa 0.76–0.86.

**Table 4:** Evaluation summaries for the prediction on individual biomes on the test set for the Random Forests classifier.

Overall accuracy 0.86		Predicted biomes					Evaluation metrics			
Overall kappa 0.75		DXS	FGS	MGS	TSMBF	TSGSS	Recall	Precision	F1	Kappa
**Observed biomes**	Deserts and Xeric Shrublands	**22**	0	1	0	1	0.73	0.92	0.81	0.76
	Flooded Grasslands and Savannas	2	**0**	0	0	0	0.00	0.00	-	-
	Montane Grasslands and Shrublands	1	0	**10**	1	0	0.77	0.83	0.80	0.77
	Tropical and Subtropical Moist Broadleaf Forests	0	0	2	**27**	2	0.93	0.87	0.90	0.86
	Tropical and Subtropical Grasslands, Savannas, and Shrublands	5	0	0	1	**36**	** **	0.92	0.86	0.89	0.83

Number of correct predictions run diagonally and are highlighted in bold. Recall for for each predicted vegetation type is calculated as the weighted number of correct predictions for a given known vegetation type. Precision for each predicted vegetation type is calculated as the weighted proportion of correctly classified vegetation unit to the sum of all predictions.

**Fig 3:**
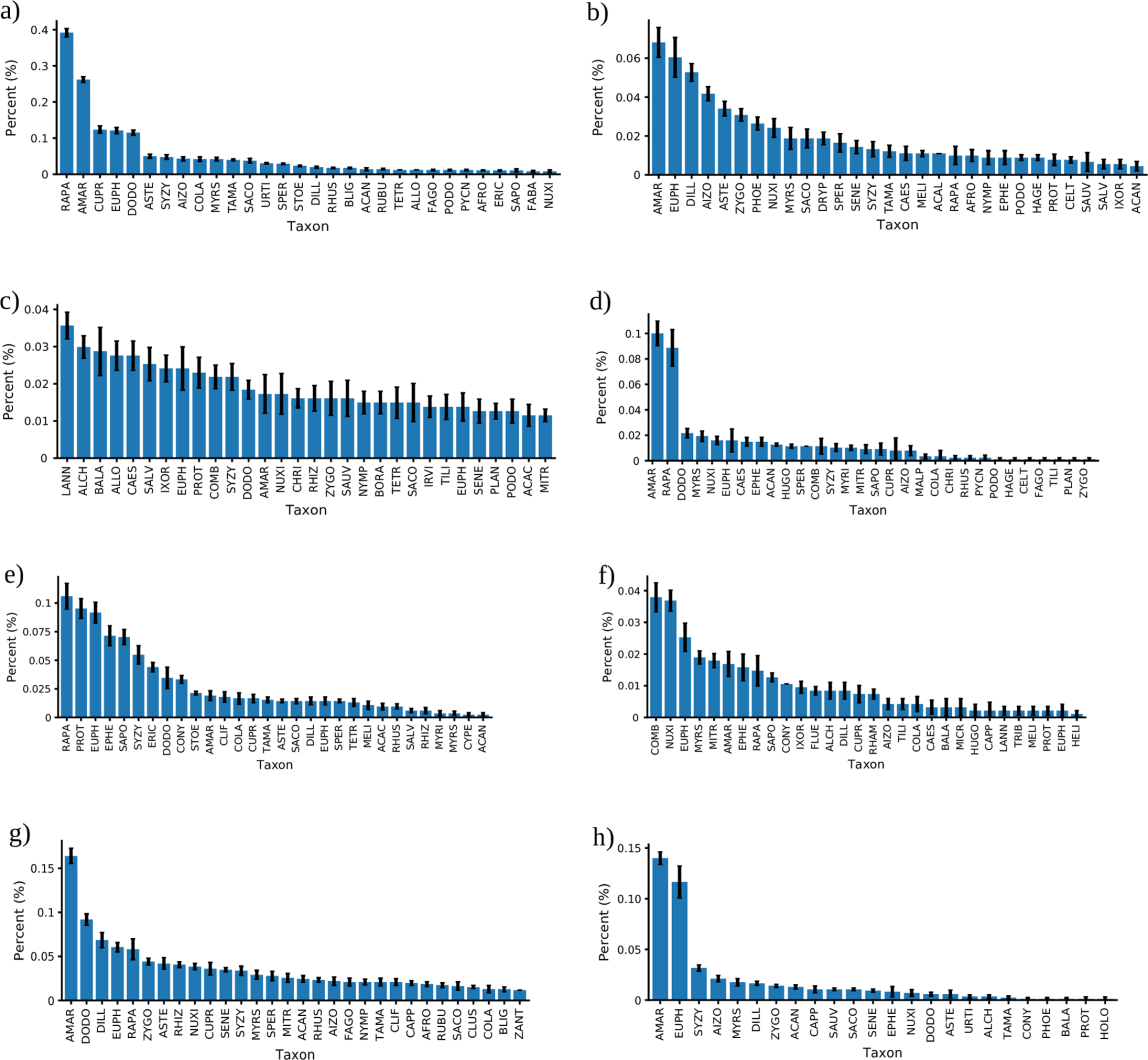
Mean decrease in accuracy (MDA) calculated for the machine learning classifiers identifying pollen taxa that contribute to high predictions. a) Linear Discriminant Analysis, b) Logistic Regression, c) Naïve Bayes, d) K-Nearest Neighbours, e) Classification Decision Tree, f) Random Forest, g) Support Vector Machine, h) Neural Network. Error bars are standard error of the mean. For each model the most important 30 taxa are plotted. Abbreviations of pollen taxon names along with their MDA percentages may be found in S2 Table.

The contribution of the 30 most important pollen taxa to the overall prediction accuracy of each model is shown in Fig 3 (for full taxon names see S3 Table). Amaranthaceae and Euphorbiaceae are the most frequent taxa chosen by the models to a varying degree of importance. Amaranthaceae is the most important taxon in KNN, LR, NN, SVM classifiers and is chosen as one of the top three taxa by the LDA model. Euphorbiaceae is an important taxon for LR, LDA, CDT, NN, and RF classifiers. *Rapanae* spp (Primulaceae) is the most important taxon for accurate predictions in the LDA and CDT models and contributes highly to the KNN classifier. Other important taxa include Dodonaea and Dilleniaceae. For the RF model the three most important taxa are Combretaceae, *Nuxia*, and Euphorbiaceae (Fig 3F).

The PFT model [17 achieves the overall accuracy of 0.71 and overall kappa of 0.63 (Table 5). Evaluation metrics for individual biomes range between 0.23–0.9 for recall, 0.16–1 for precision, 0.27–0.75 for F1, and 0.32–0.73 for kappa statistic.

**Table 5:** Evaluation metrics calculated for the PFT-based biome model (Jolly et al., 1998, Table 4).

Overall accuracy 0.71		Predicted biomes								Evaluation metrics			
Overall kappa 0.63		DESE	STEP	SAVA	XERO	WAMF	TDFO	TSFO	TRFO	Recall	Precision	F1	Kappa
**Observed biomes**	Desert	**5**	7	0	1	0	0	0	0	0.38	1.00	0.56	0.55
	Steppe	0	**126**	25	14	0	1	0	0	0.76	0.16	0.27	0.70
	Savanna	0	27	**206**	25	3	7	3	0	0.76	0.74	0.75	0.64
	Temperate Xerophytic Woods/Scrub	0	2	3	**98**	6	0	0	0	0.90	0.49	0.63	0.57
	Warm Mixed Forest	0	2	2	54	**140**	1	0	0	0.70	0.88	0.78	0.73
	Tropical Dry Forest	0	3	41	8	7	**21**	8	2	0.23	0.70	0.35	0.32
	Tropical Seasonal Forest	0	0	2	0	4	0	**32**	0	0.84	0.63	0.72	0.70
	Tropical Rain Forest	0	0	0	0	0	0	8	**13**	** **	0.62	0.87	0.72	0.72

Number of correct predictions run diagonally and are highlighted in bold. Recall and precision are calculated as in Table 4.

## Discussion

### Biome predictions

Pollen is a direct and quantitative link to the vegetation that produced it. Thus, under ideal conditions we would expect to predict biomes from pollen to a high degree of accuracy. However, pollen analysis has sources of loss and error that reduce accuracy and precision. First, pollen preservation potential is related to dispersal syndrome, with the majority of deposited grains derived from wind-pollinated vegetation [91]. Second, pollen preserves under specific conditions; water-logged and anaerobic conditions, such as those characteristic of lake sediments, peats, and swamps, are ideal for pollen preservation [92]. These contexts accumulate pollen from a broad catchment source and provide the most representative signature of vegetation. Where preservation conditions are unfavorable, pollen may be sourced from other deposits, e.g. snow [93,94], pack rat middens [95–97], and hyena scat [98]. Third, not all pollen is created equal. Taphonomic processes, such as oxidation [99–101], microbial activity [102–104], wet-dry cycles [105,106,102], and changes in pH [107], may lead to differential destruction of pollen grains varying in exine thickness and other physical properties. Fourth, pollen grains may be lost during the process of laboratory preparation of samples [92]. Lastly, microscopic identification of pollen grains by humans is highly dependent on various conditions, including expertise level and psychological state [108].

Yet, despite these potential sources of loss and error we are able to successfully (as assessed by Kappa) predict four out of five biomes from pollen data using supervised machine learning and specifically the Random Forest classifier. Biomes predicted to a very high level of accuracy and precision are the Tropical and Subtropical Moist Broadleaf Forests and the Tropical and Subtropical Grasslands, Savannas, and Shrublands (Table 4). Furthermore, the Deserts and Xeric Shrublands, and Montane Grasslands and Shrublands biomes are also predicted accurately and precisely. All of these biomes are well represented in the modern pollen dataset (Table 1). In contrast, our model performs poorly in predicting the Flooded Grasslands and Savannas (FGS) biome (Table 4).

However, this low prediction on FGS is not unique to the RF classifier. With the exception of the KNN classifier, none of the models are able to accurately predict FGS. Factors contributing to this poor performance may relate to sampling noise at the pollen level. The FGS pollen assemblages are dominated by cosmopolitan pollen types, primarily Poaceae and Cypereaceae. Furthermore, there are fewer total pollen taxa present in the FGS biome assemblages as compared to the other four biomes. Although pollen taxa specific to the FGS biome are present, including aquatics such as *Typha* and *Nymphaea*, their signal may be diluted by the cosmopolitan species.

More likely, the poor performance of the RF model on the FGS biome is explained by sampling noise at the biome level; the FGS biome class is under-represented in the original pollen dataset (21 sites). In addition, the dataset is further split into training and testing sets in the 9:1 ratio. Splitting data into training and testing sets is necessary for developing an accurate and robust model, but it also results in two complications. Firstly, the small training sample of the FGS biome means that learning of the relationships between biome and pollen data is compromised. Secondly, the test set contains only two examples of the FGS biome. Thus, test performance can be much more prone to the influence of sampling error. For instance, if examples in the test set happen to be harder to classify (i.e. represent transitional vegetation), the model’s performance will decrease. Thus, adequate representation of biomes in the pollen data is essential in building a robust and reliable predictive model.

### Significant taxa

In Africa seventeen modern pollen indicator taxa were identified by plotting their relative abundances against annual temperature and precipitation [24]. The majority of these indicators represent lower taxonomic ranks of species or genera. In contrast, the majority of the top five most important pollen taxa with respect to the RF model represent family ranks. These differences are expected as the two studies investigate different questions. The previous study attempted to identify potential pollen indicators for quantitative reconstruction of temperature and precipitation. Thus, lower taxonomic ranks emerge as indicators as they are confined to smaller geographic ranges than taxa ranked at the Family level. Plant families tend to extend over wider geographic space and longer environmental gradients than genera or species, and thus are less likely to be useful in quantitative paleoclimatic reconstructions.

However, many pollen datasets contain large numbers of taxa at the Family level. Our analysis shows that this limitation of many pollen datasets has a smaller impact on the categorical classification of assemblages into biomes. For predicting biomes, higher taxonomic rank that encompass a much larger climatic range are more useful suggesting that this approach works well for very long environmental gradients. In our Random Forest model important pollen taxa are indicated by the mean decrease in accuracy (MDA); the higher the MDA for a given taxon, the more important it is to the model's prediction. The top three taxa contributing to the high prediction of the RF model are Combretaceae, *Nuxia*, and Euphorbiaceae (Fig 1F).

Exclusion of Combretaceae leads to a 4% decrease in the Random Forest model predictive performance. Combretaceae is a family of flowering plants including trees, shrubs, mangroves, and lianas. The family is distributed across the globe with the highest species richness in the tropical and subtropical regions of the Old World particularly rainforest, savannah, woodland, and mangrove ecosystems [109,110]. As an indicator taxon in the pollen record, Combretaceae is linked to mesic type savanna [111, 112] and dry bushveld [113]. Given its large range, Combretaceae is an important component of diverse biomes such as savanna, xerophytic scrub, and various tropical forest types including dry, seasonal, and rain forests [15]. As such, Combretaceae is important to the RF model predictions of the Tropical and Subtropical Moist Broadleaf Forests, as well as Tropical and Subtropical Grasslands, Savannas and Shrublands biomes and plays a role in modeling the Deserts and Xeric Shrublands biome.

Exclusion of *Nuxia* leads to a 4% decrease in the RF model predictive performance. The *Nuxia* genus in the Stilbaceae family [114,115] of flowering plants is found in tropical Africa and is particularly characteristic of African and Madagascar montane forests. This taxon is also present in open forests and scrub, though rarely in savannas [116]. *Nuxia* is often indicative of afromontane vegetation [16] and warm temperatures [117]. Thus, for our model this genus holds significance to the prediction of the Montane Grasslands and Shrublands, the Tropical and Subtropical Grasslands, Savannas and Shrublands, and the Tropical and Subtropical Moist Broadleaf Forests.

Euphorbiaceae is a large family of flowering plants that includes herbs, shrubs, and succulents. Within the family, the Euphorbia genus is one of the most diverse and largest in the world with majority of the species endemic to Africa and Madagascar where they occur in variety of environments [118]. In the pollen record, both Euphorbiaceae and Euphorbia often reflect dry conditions [119] and are often interpreted as indicative of semiarid conditions such as those associated with southern African Succulent Karoo biome [120,121]. Exclusion of Euphorbiaceae from the RF classifier leads to a 2.5% decrease in the model's performance. This high importance of Euphorbiaceae to the model’s predictions combined with its modern broad geographical distribution indicate this family to be a significant taxon for discriminating between extreme biomes, i.e. arid vs tropical. Furthermore, considering their status as an indicator taxa of semi-arid conditions, we interpret Euphorbiaceae as important in determining arid and semi-arid environment represented by the Desert and Xeric Shrublands biome.

Nevertheless, interpretations of importance variables must be made with caution. Feature importances provide insights about how individual pollen taxa affect the predictive power of each trained model. However, these measures do not enable any inferences about the relationship between pollen features and prediction of individual biome classes or the relationships between pollen taxa with one another. Furthermore, pollen taxa with low feature importance values should not be discounted as unimportant to prediction as low features values may suggest that the model placed more weight on a correlated feature. For instance, if taxon A and taxon B are highly correlated they carry roughly the same amount of information and the model may place importance on only one of them without compromising performance.

The Random Forest model presented here may be compared to the biomization method on the bases of statistical metrics. However, the PFT-based biome model for Africa does not provide directly equivalent statistical evaluations [17]. The PFT model scores lower than the RF model (Table 5). However, there are several reasons precluding direct comparisons between the two approaches. For direct comparisons between the two models, the RF classifier must be applied to the pollen data originally labeled with the PFT-based biomes. Although the same pollen data were used, the biome label assignments used different versions of the same classification systems; while the PFT model uses an older version of the classification system [122], our machine learning model uses the most recent 2001 version [26]. For instance, in the 1983 version there is no equivalent for Tropical and Subtropical Moist Broadleaf Forest present in the 2001 version. Furthermore, the number of biome labels is different; the PFT model has seven biome classes, while the RF model was trained on five classes.

To illustrate the importance of a consistent classification system, consider the following thought experiment. Take the same data our RF model is trained on. Then, randomly shuffle the biome assignments and train a new RF model on the shuffled data. Since there is no longer any correlation between pollen data and biome assignments, the performance of the model trained on randomly assigned biome labels is expected to be much lower. When labels are shuffled randomly this presents a harder learning problem. Although this is a contrived example, it demonstrates that classification results are not purely a function of the model or the input data (i.e. pollen counts), but are strongly influenced by the label assignment.

### Paleoenvironmental reconstruction

The proof-of-concept Random Forests classifier, validated and tested on modern pollen data, has the potential for highly accurate predictions of past biomes and awaits application to fossil pollen sequences for prediction of past biomes. In Africa, the RF model may be used to investigate events on both long and short time scales, such as the late Pleistocene arid events [123–125] or more recent and abrupt climatic anomalies like the African Humid Period (AHP) [126,127,16]. The AHP has been linked to the greening of Sahara via empirical [128] and modeling approaches [129,130]. Previous biome modeling studies predict northward shift of tropical rain forest around 11–9 ka and a reduction of the desert biome at the termination of the AHP [131,15,17]. The Random Forest model may provide additional insights by quantifying the probability of these biomes occurring at discrete times. Similarly, the progression and magnitude of the AHP over the African continent may be constrained using our probabilistic model aiding, for example, in the understanding of the spatio-temporal extent of the AHP and its impact on higher latitudes of southern Africa [132–135].

Our model may be applied to other paleo-related research areas The high capacity of our machine learning model for discerning hidden and non-linear patterns in complex datasets may reveal new insights and generate new hypotheses in paleosciences. The model may also have potential application in archeology as various aspects of human evolution have been linked to climate and resource availability including occupation patterns [136], agriculture and pastoralism [137,138], and the rise and fall of ancient civilizations [139,140]. The model may be applied to prediction and modeling of smaller scale vegetation units using regional vegetation classification to allow for higher resolution picture of past shifts in regional vegetation cover providing valuable information on regional drivers of climate change. Lastly, the Random Forest algorithm may find application for regressions problems in palynology as RF-based quantitative estimates of climate variables from pollen data are a relatively new approach [141].

### Advantages of machine learning approaches to pollen-based biome prediction and modeling

Our new machine learning approach using the Random Forest algorithm predicts biome types to a high level of accuracy. The RF model provides improvements for biome predictions from fossil pollen sequences by incorporating more criteria. Both the indicator species and the biomization methods rely on the reduction of information from taxonomically rich pollen datasets to only few taxa. These approaches are well-founded given the low signal-to-noise ratio in pollen datasets and high computational demands necessary to analyze these complex datasets. However, it is possible that the reductionist assumptions may not capture the entirety of valuable information available in the pollen data. Novel machine learning methods and higher computational power permit a more complete analysis of complex and noisy data such as pollen data.

Furthermore, the application of various machine learning models may shed light on the nature of the relationship between pollen and biome types. We investigated a number of popular parametric, non-parametric and semi-parametric models, each representative of specific set of assumptions. In our analysis, a definite non-linear component is apparent as the Random Forest classifier achieves the highest predictive performance on the prediction task. Yet, linear assumptions appear to hold significant validity with regards to the relationship between pollen and biome types. The Linear Regression model makes strong linear assumptions and yet achieves the second highest performance on the prediction task. Likewise, the simplified assumptions of the Naïve Bayes model, that posits no interaction between pollen taxa, result in comparatively high prediction. Furthermore, for the NB model the probability distribution chosen for pollen features was Bernoulli (i.e. presence/absence). Thus, even when the proportion of individual pollen features is ignored, the classification and prediction of biomes using only presence/absence proxy data is possible to a relatively high degree (Table 3).

### Limitations of machine learning approaches to pollen-based biome prediction and modeling

**Fig 4:**
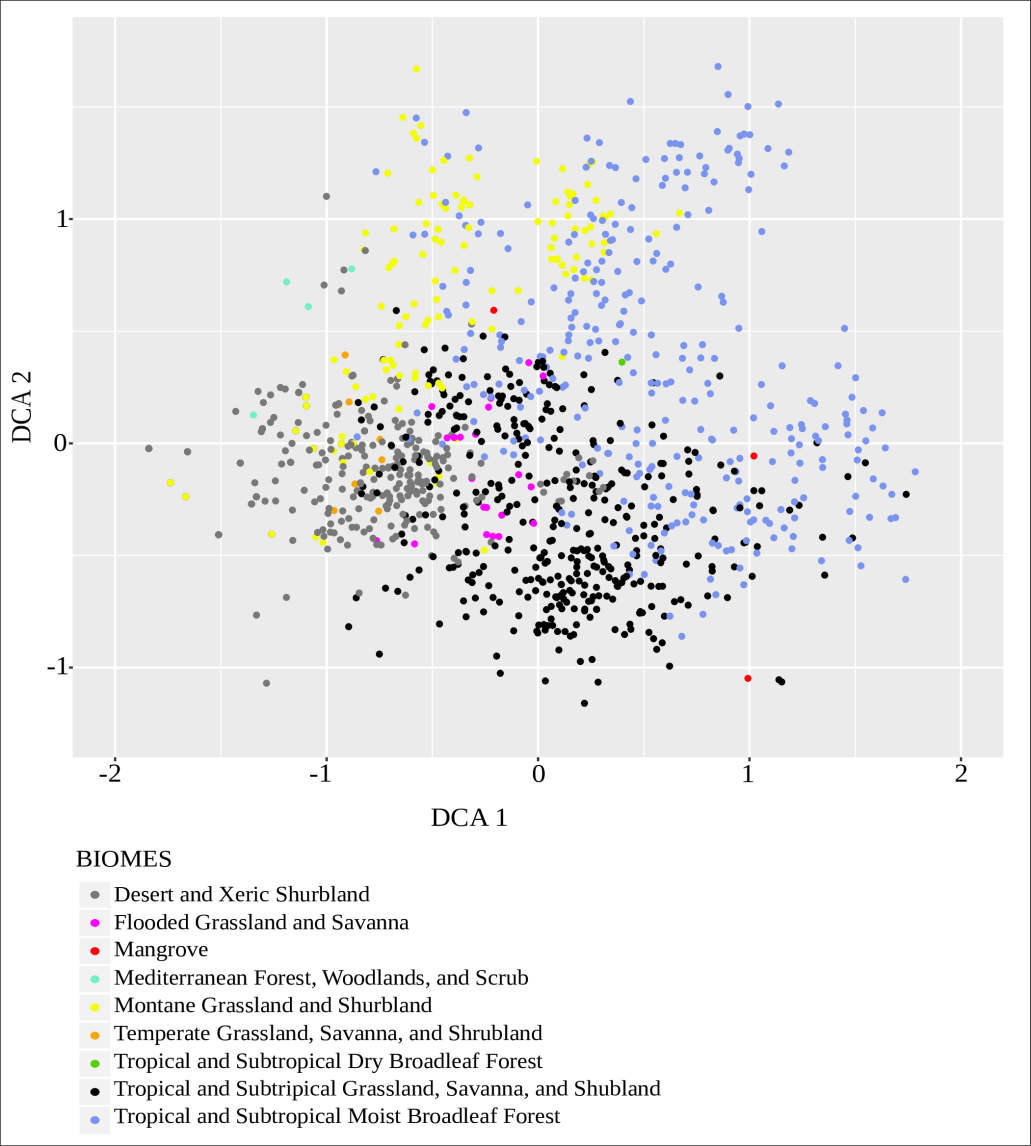
Detrended correspondence analysis (DCA) of the modern pollen assemblages color-coded by biome type (Olson et al., 2001).

Our pollen-based predictive biome model is an analogue method. As such, its performance relies on robust modern pollen dataset for training purposes. Thus, our method is not applicable for regions of the world where modern datasets are unavailable. Where biomes are well represented in the modern pollen dataset (Fig 4), the performance of our model is high as indicated by high scores on the evaluation metrics attained for both the overall model prediction (Table 3) and individual biome predictions (Table 4) suggesting it to be a robust and reliable classifier. For reconstructions of vegetation from Deep Time circumstances our model’s performance would be dependant upon assumptions about past environmental conditions that may differ from modern environments. In such cases, the most useful information about past environmental conditions may be gained by combining our method with other available approaches.

## Future work

Future work is needed to establish statistical comparability between the results of our Random Forest classifier and the biomization method for African biomes. This may be achieved by using our Random Forest algorithm on the PFT-based biome labels from [17]. Alternatively, the biomization method may be applied to our data labeled with our biome classes.

Another area for potential improvement may be labels used for biome assignment. As classification results are influenced by label assignment, more accurate labels for vegetation classes would result in more accurate model and predictions. For our biome labels we use an inclusive classification system that places biota at the core of the concept and encompasses distinct assemblages of species [26]. However, the label assignments could be improved by using high resolution satellite data for most current vegetation distribution and classification.

Moreover, the performance of Neural Networks on the task of biome prediction from pollen data may be improved. Neural Networks are universal function approximators, theoretically able to learn any function [142]. Our hypothesis that the NN model was expected to be one of the highest preforming models was met. However, here only a simple feed forward neural network with one hidden layer [21] was used to predict biomes from pollen data. This model is relatively slow to optimize (S1 Table) but achieves high performance (Table 3) in predicting biome classes from pollen data. However, state-of-the-art classification using NN models is achieved for sequentiality and spatially structured data such language translations [143] and image recognition [144]. However, pollen data is neither sequentially nor spatially structured. A new self-normalizing NN model [145] has recently been developed for application to broader classification problems and may achieve higher performance than the feed forward NN used in our analysis.

## Conclusions

We develop a new robust model for modern biome predictions using vegetation proxy data via a supervised classification approach. By testing and validating various machine learning classifiers we identify the Random Forest algorithm as the highest performing model. The model may be now applied to fossil pollen sequences for probabilistic reconstructions of past biomes. Thus, our model has the potential to improve understanding of spatial and temporal distribution of past vegetation.

## Supporting information

S1 Table Time requirement for hyper-parameter optimization for the validation set. Hyper-parameters were fitted to 10 folds for each of 50 candidates, totaling 500 fits. Acronyms denote LDA for Linear Discriminant Analysis, SVM for Support Vector Machines, NN for Neural Networks, RF for Random Forest, LR for Logistic Regression, NB for Naïve Bayes, CDT for Classifivation Decision Tree, and KNN for K-Nearest Neighbors.
(XLS)

S2 Table List of biome assignments (Olson et al., 2001) to modern pollen data (Gajewski et al. 2002). Latitude (Lat) and longitude (Long) values were rounded to the nearest two decimal points. The column “Context” refers to the deposit type a pollen sample was collected from. The collumn “Symbol” refers to a color-coded position of a pollen sample on map in Fig 2.
(XLS)

S3 Table Complete list of mean decrease in accuracy (MDA) metrics for all pollen taxa calculated for each model. Acronyms denote LDA for Linear Discriminant Analysis, SVM for Support Vector Machines, NN for Neural Networks, RF for Random Forest, LR for Logistic Regression, NB for Naïve Bayes, CDT for Classifivation Decision Tree, and KNN for K-Nearest Neighbors.
(XLSX)

S1 Python Code Documented Python code used to train and evaluate the eight statistical and machine learning classification models for the task of biome prediction using pollen data. Documentation in the code consists of comments (#) and docstrings (' ' '). Comments explain that portion of the code and are placed immediately before the section of code they refer to. Docstrings provide a detailed description of a function and are placed after the function is defined.
(DOCX)
