# Supplementary material for: Predictive pollen-based biome modeling using machine learning
Source: PLoS One. 2018 Aug 23;13(8):e0202214. doi: 10.1371/journal.pone.0202214 (PMC6122137; doi:10.1371/journal.pone.0202214)
Supplement: S1 Python Code — Documentation in the code consists of comments (#) and docstrings (' ' '). Comments explain that portion of the code and are placed immediately before the section of code they refer to. Docstrings provide a detailed description of a function and are placed after the function is defined. (DOCX) [file pone.0202214.s004.docx]

**S3 Phyton code.** **Documented Python code used to train and evaluate the eight statistical and machine learning classification models for the task of biome prediction using pollen data.** Documentation in the code consists of comments (#) and docstrings (' ' '). Comments explain that portion of the code and are placed immediately before the section of code they refer to. Docstrings provide a detailed description of a function and are placed after the function is defined.

import os

import numpy as np

import pandas as pd

import matplotlib.pyplot as plt

import scipy

from collections import defaultdict

from functools import partial

from sklearn.model_selection import StratifiedKFold, RandomizedSearchCV

from sklearn.model_selection import train_test_split

from sklearn.linear_model import LogisticRegression

from sklearn.ensemble import RandomForestClassifier

from sklearn.svm import SVC

from sklearn.discriminant_analysis import LinearDiscriminantAnalysis

from sklearn.neural_network import MLPClassifier

from sklearn.pipeline import Pipeline

from sklearn import metrics

from sklearn.naive_bayes import GaussianNB, MultinomialNB, BernoulliNB

from sklearn.neighbors import KNeighborsClassifier

from sklearn.tree import DecisionTreeClassifier

from scipy.stats import randint, uniform

from sklearn.externals import joblib

# Global variables

# Set random seed for repeatability

seed = 42

out_dir = 'model_output'

n_folds = 10

n_iter_search = 50

def exclude_rare_taxa(x, threshold=3):

to_keep = (x > threshold).sum(axis=0) != 0

return x[:, to_keep]

# Define functions for evaluation metrics

f1_micro = partial(metrics.f1_score, average='micro')

f1_macro = partial(metrics.f1_score, average='macro')

f1_weighted = partial(metrics.f1_score, average='weighted')

precision_micro = partial(metrics.precision_score, average='micro')

precision_macro = partial(metrics.precision_score, average='macro')

precision_weighted = partial(metrics.precision_score, average='weighted')

recall_micro = partial(metrics.recall_score, average='micro')

recall_macro = partial(metrics.recall_score, average='macro')

recall_weighted = partial(metrics.recall_score, average='weighted')

def train_and_validate(pipe, x_train, y_train, x_test, y_test, test_metrics,

scores):

'''

Return trained pipeline and confusion matrix. Also populate test_metrics

dictionary and scores dictionary.

'''

pipe.fit(x_train, y_train)

y_proba = pipe.predict_proba(x_test)

y_pred = y_proba.argmax(axis=1)

# Calculate confusion matrix on test set data

confusion_mat = metrics.confusion_matrix(y_test, y_pred)

for name, metric_fn in [('accuracy', metrics.accuracy_score),

('kappa', metrics.cohen_kappa_score),

('f1_macro', f1_macro),

('f1_micro', f1_micro),

('f1_weighted', f1_weighted),

('precision_macro', precision_macro),

('precision_micro', precision_micro),

('precision_weighted', precision_weighted),

('recall_macro', recall_macro),

('recall_micro', recall_micro),

('recall_weighted', recall_weighted)]:

score = metric_fn(y_test, y_pred)

test_metrics[name].append(score)

# Calculate mean decrease in accuracy over 10 permutations

acc = metrics.accuracy_score(y_test, y_pred)

names = list(pollen_only)

np.random.seed(seed)

for i in range(x.shape[1]):

# shuffle 10 times

for j in range(10):

x_test_copy = x_test.copy()

np.random.shuffle(x_test_copy[:, i])

shuff_acc = metrics.accuracy_score(

y_test,

pipe.predict_proba(x_test_copy).argmax(axis=1))

scores[names[i]].append((acc - shuff_acc) / acc)

return pipe, confusion_mat

if __name__ == '__main__':

# Load the csv using pandas

df = pd.read_csv('OlsenVeg.csv')

# Filter rows for biomes that occur in less than 10 sites

df = df.groupby("BIO_N").filter(lambda x: len(x) >= 10)

# Remove non-pollen columns; all rows, starting at 9th column onwards

pollen_only = df.iloc[:, 9:]

# Convert data to a matrix

pollen_matrix = pollen_only.as_matrix()

# Rename input data (pollen) to 'x', output data (biomes) to 'y'

x = pollen_matrix.copy()

cat = pd.Categorical(df.BIO_N)

# Convert category names to numbers

y = cat.codes

# Store names of the categories (i.e. biomes)

labels = cat.categories

x = exclude_rare_taxa(x)

# Scale values so between [0,1]s

x /= 100

print("Max: {}; Min: {}".format(x.max(), x.min()))

# Define all classifiers and hyper-parameters

classifiers = [

{

'model': LogisticRegression(random_state=seed),

'param_dist': {

'class_weight': [None, 'balanced'],

'fit_intercept': [True, False],

'C': uniform(0, 1000),

'solver': ['lbfgs'],

'multi_class': ['ovr', 'multinomial'],

},

},

{

'model': RandomForestClassifier(

random_state=seed),

'param_dist': {

'n_estimators': randint(10, 200),

'criterion': ['gini', 'entropy'],

'max_features': ['auto', 'sqrt', 'log2', None],

'min_samples_split': uniform(0.0, 1),

'class_weight': ['balanced', 'balanced_subsample'],

},

},

{

'model': MLPClassifier(random_state=seed),

'param_dist': {

'hidden_layer_sizes': [(50,), (100,), (200,)],

'alpha': uniform(0, 0.1),

'activation': ['logistic', 'tanh', 'relu'],

'solver': ['adam'],

'batch_size': [32, 64, 128],

'learning_rate': ['constant', 'adaptive'],

'learning_rate_init': [0.0001],

'max_iter': randint(20, 200),

},

},

{

'model': LinearDiscriminantAnalysis(),

'param_dist': {

'solver': ['svd', 'eigen', 'lsqr'],

'n_components': randint(1, 5),

},

},

{

'model': GaussianNB(),

'param_dist': None,

},

{

'model': MultinomialNB(),

'param_dist': {

'fit_prior': [True, False],

'alpha': uniform(0, 1),

},

},

{

'model': BernoulliNB(),

'param_dist': {

'fit_prior': [True, False],

'alpha': uniform(0, 1),

},

},

{

'model': KNeighborsClassifier(),

'param_dist': {

'n_neighbors': randint(1, 10),

'weights': ['uniform', 'distance'],

'algorithm': ['ball_tree', 'kd_tree', 'brute'],

'p': randint(1, 4),

},

},

{

'model': DecisionTreeClassifier(random_state=seed),

'param_dist': {

'criterion': ['gini', 'entropy'],

'splitter': ['best', 'random'],

'max_features': ['auto', 'sqrt', 'log2', None],

'min_samples_split': uniform(0.0, 1),

'class_weight': ['balanced', None],

},

},

{

'model': SVC(probability=True, random_state=seed),

'param_dist': {

'C': uniform(0.001, 100),

'gamma': uniform(0.001, 1000),

'kernel': ['rbf', 'poly', 'sigmoid'],

'degree': randint(1, 4),

},

},

]

# Create dictionaries for storing models and results

all_metrics = {}

all_models = {}

feature_importance = {}

all_confusions = {}

test_metrics = {}

test_confusion = {}

# Create the output directory

if not os.path.exists(out_dir):

os.makedirs(out_dir)

# Split into train/test sets. Training set = 90%

x, x_test, y, y_test = train_test_split(x, y,

test_size=0.1,

random_state=seed,

stratify=y)

# Loop over all models

for clf_dict in classifiers:

clf = clf_dict['model']

param_dist = clf_dict['param_dist']

print("#" * 80)

print("Fitting: {}".format(clf))

# Use StratifiedKFold for k-fold data splitting when there are

# large class imbalances

folds = StratifiedKFold(

n_splits=n_folds,

shuffle=True,

random_state=seed)

if param_dist is not None:

# Only do hyperparameter search if param_dist is not 'None',

# i.e. if the model has no hyper-parameters to search over

random_search = RandomizedSearchCV(

clf,

cv=folds,

verbose=1,

n_jobs=4,

param_distributions=param_dist,

n_iter=n_iter_search,

random_state=seed)

# Do hyperparameter search

random_search.fit(x, y)

print("Best parameters:")

print(random_search.best_params_)

clf.set_params(**random_search.best_params_)

test_metrics = defaultdict(list)

models = []

scores = defaultdict(list)

confusion_mat = []

# Train on all training data and get test performance

pipe = Pipeline(steps=[

('clf', clf),

])

pipe, cm = train_and_validate(pipe, x, y, x_test, y_test,

test_metrics, scores)

models.append(pipe)

confusion_mat.append(cm)

# Display evaluation metrics

print("\nSummary on test set:")

print("Model: {}".format(clf.__class__.__name__))

print("#" * 50)

for metric in sorted(test_metrics.keys()):

m = test_metrics[metric]

print("{:<24}: {:.5f} ({:.5f})".format(

metric,

np.mean(m),

np.std(m)))

# Save model and evaluation metrics for later

all_metrics[clf.__class__.__name__] = test_metrics

all_models[clf.__class__.__name__] = models

feature_importance[clf.__class__.__name__] = scores

all_confusions[clf.__class__.__name__] = np.stack(confusion_mat)

# Save model and evaluation metrics to disk

dump = {

'test_metrics': test_metrics,

'models': models,

'scores': scores,

}

joblib.dump(dump, '{}/{}_dump.pkl'.format(

out_dir, clf.__class__.__name__))

# Write metrics for all models out to csv

df_metrics = {}

for model in all_metrics:

val_metrics = all_metrics[model]

data = []

index = []

for metric in sorted(val_metrics.keys()):

m = val_metrics[metric]

data.append(np.mean(m))

index.append(metric + "_mean")

data.append(np.std(m))

index.append(metric + "_std")

df_metrics[model] = pd.Series(data, index=index)

df_metrics = pd.DataFrame(df_metrics).transpose()

df_metrics.to_csv("{}/results.csv".format(out_dir))

# Write confusion matrices, precision, recall and f1 for all models out

# to csv

for model in all_confusions:

df_conf = pd.DataFrame(all_confusions[model].mean(axis=0))

df_conf.columns = labels

# Calculate class based precision/recall

conf = df_conf.values

recall = conf.diagonal() / conf.sum(axis=0)

precision = conf.diagonal() / conf.sum(axis=1)

f1 = 2 * (precision * recall) / (precision + recall)

# Calculate Cohen's Kappa

prob_mat = conf / conf.sum()

row_sum = prob_mat.sum(axis=0)

col_sum = prob_mat.sum(axis=1)

kappa = ((

prob_mat.diagonal() - row_sum * col_sum) /

((row_sum + col_sum) / 2 - row_sum * col_sum))

df_conf['recall'] = pd.Series(recall, df_conf.index)

df_conf['precision'] = pd.Series(precision, df_conf.index)

df_conf['f1'] = pd.Series(f1, df_conf.index)

df_conf['kappa'] = pd.Series(kappa, df_conf.index)

df_conf.to_csv("{}/{}_confusion.csv".format(out_dir, model))

# Plot and output to csv feature importances for all models

for model in feature_importance:

mean_decrease_in_accuracy = []

for k in feature_importance[model].keys():

scores = feature_importance[model][k]

mean_score = np.mean(scores)

sem_score = scipy.stats.sem(scores)

if mean_score > 0:

mean_decrease_in_accuracy.append(

(k, mean_score, sem_score))

mean_decrease_in_accuracy.sort(key=lambda x: x[1], reverse=True)

# Plot feature importances and write to pdf

names, scores, sems = zip(*mean_decrease_in_accuracy[:30])

plt.clf()

plt.bar(range(len(names)), scores, yerr=sems, capsize=2)

plt.xticks(

range(len(names)),

[name[:4] for name in names],

rotation='vertical',

fontsize=10)

plt.ylabel("Percent (%)", fontsize=12, labelpad=12)

plt.ylim(ymin=0)

plt.xlabel("Taxon", fontsize=12)

plt.gca().spines['top'].set_visible(False)

plt.gca().spines['right'].set_visible(False)

plt.xlim((-0.6, len(names) - 0.4))

plt.tight_layout(rect=[0, 0.05, 1, 1])

fig = plt.gcf()

fig.set_size_inches(7, 3)

fig.savefig(

'{}/{}_mean_decrease_accuracy.pdf'.format(out_dir, model),

dpi=300)

# Write feature importances to csv

mda_series = pd.Series(scores, index=names)

mda_series.to_csv(

'{}/{}_mean_decrease.csv'.format(out_dir, model))
